# 28201 A cross-sectional study of dietary patterns and nutrient intakes in the oldest old

**DOI:** 10.1017/cts.2021.708

**Published:** 2021-03-30

**Authors:** Ashley C. Flores, Yi-Hsuan Liu, Xiang Gao, G. Craig Wood, Brian A. Irving, Christopher D. Still, Gordon L. Jensen, Diane C. Mitchell

**Affiliations:** 1 The Pennsylvania State University; 2 Geisinger Health System; 3 Pennington Biomedical Research Center; 4 University of Vermont

## Abstract

ABSTRACT IMPACT: Understanding dietary patterns and nutrient intakes of the aging population may help address concerns and dietary guidelines regarding their nutritional needs. OBJECTIVES/GOALS: The objective of this study is to test the hypothesis that a healthy dietary pattern in the oldest old (aged 80 years and older) is related to greater compliance with dietary recommendations and better nutrient intake profiles. METHODS/STUDY POPULATION: We conducted a cross-sectional study of 122 participants aged 82 to 97 years old from the Geisinger Rural Aging Study (GRAS) cohort in rural Pennsylvania (n = 56 men and 66 women). The main outcome measures of the investigation were the daily nutrient intakes and food group intakes evaluated from the average of three 24-hour dietary recalls. The dietary patterns were determined by cluster analysis from 28 food groups. Diet quality and adherence to the Dietary Guidelines for Americans was assessed by the Healthy Eating Index (HEI)-2015 and the Dietary Screening Tool (DST). Recommended intakes were determined by the Recommended Dietary Allowances (RDAs) or Adequate Intakes (AIs). RESULTS/ANTICIPATED RESULTS: Less than 50% of participants met the dietary recommended intakes for vitamins D, E, K, B6, dietary fiber, zinc, potassium, and calcium. The more-nutrient-dense cluster was characterized by higher intakes of fruits and vegetables. The less-nutrient-dense cluster was characterized by higher intakes of foods including desserts and sweets. After adjusting for age, sex, and energy intake, participants in the more-nutrient-dense dietary pattern had a higher intake of vitamins A, D, K, C, fiber, and potassium (p < 0.05 for all). After adjusting for age and sex, participants in the more-nutrient-dense pattern had better diet quality assessed by the (HEI)-2015 (p < 0.001) and DST (p = 0.006). DISCUSSION/SIGNIFICANCE OF FINDINGS: Among the oldest old, many participants were found to have nutrient intakes lower than the recommended levels for fundamental nutrients suggesting that dietary guidance in addition to a dietary pattern more aligned with dietary guidelines may be beneficial for supporting healthy aging.

